# Perineural Injection for Treatment of Root-Signature Signs Associated with Lateralized Disk Material in Five Dogs (2009–2013)

**DOI:** 10.3389/fvets.2016.00001

**Published:** 2016-01-27

**Authors:** Sarah Giambuzzi, Theresa Pancotto, Jeffrey Ruth

**Affiliations:** ^1^Lake Forest Animal Hospital, Forest, VA, USA; ^2^Department of Small Animal Clinical Sciences, Virginia Maryland College of Veterinary Medicine, Blacksburg, VA, USA

**Keywords:** perineural, root-signature, intervertebral disk disease, lateralized disk, fluoroscopy

## Abstract

Intervertebral disk disease (IVDD) is common in dogs; cervical IVDD accounts for 13–25% of all cases. Ventral slot decompression provides access to ventral and centrally extruded or protruded disk material. However, procedures to remove dorsally or laterally displaced material are more difficult. This case series describes the use of perineural injection as a potential treatment option for dogs experiencing root-signature signs associated with lateralized disk material in the cervical spine. Five dogs underwent fluoroscopically guided perineural injection of methylprednisolone ± bupivacaine. Most patients experienced improvement in root-signature signs and remained pain free without the assistance of oral pain medication. These findings suggest the perineural injection of methylprednisolone ± bupivacaine represents a viable option for dogs with cervical lateralized disk material causing root-signature signs.

## Introduction

Intervertebral disk disease (IVDD) is common in dogs, especially in chondrodystrophic breeds (1). Cervical IVDD accounts for 13–25% of all cases (1). Medical management consists of cage rest, anti-inflammatory medications, muscle relaxants, and/or opioid agents. Surgical intervention is often required, due to poor response to medical therapy (2). Surgical options in the cervical region include ventral fenestration, ventral slot decompression, dorsal laminectomy, and hemilaminectomy (3–7). The ventral slot approach is the preferred method for ventrally located extrusions or protrusions (8). However, this approach may not provide access to lateralized or foraminal disk material. In such cases, dorsal laminectomy or hemilaminectomy has been used, though these procedures are more difficult, time consuming, expensive, and painful (3–7). Complications are more common with dorsal and lateral techniques compared to ventral slot and include hemorrhage, post-operative neurological deterioration, and prolonged recumbancy (7, 9, 10).

This case series describes the use of fluoroscopically guided perineural injection for dogs experiencing root-signature signs associated with lateralized disk material in the cervical spine.

## Background

Client-owned animals at the VMCVM veterinary teaching hospital that had undergone cervical, fluoroscopically guided perineural injection were retrospectively identified. For inclusion, all dogs had to have computed tomography (CT) (Toshiba Aquilon 16, Toshiba American Medical Systems Inc., Tustin, CA, USA) or magnetic resonance (Intera 1.5-T, Philips Healthcare, Eindhoven, Netherlands) findings of cervical, lateralized intervertebral disk material, and a complete medical record. Case histories are summarized in Table 1.

**Table 1:** **Summary of patient data**.

Case/signalment	Clinical signs	Rx at presentation	Imaging type	Pertinent imaging findings	Injected Rx	Oral Rx	Complications	Outcome	Follow-up
10 years CM Dachshund	1 month cervical pain, left forelimb root-signature signs	Deraxocib, tramadol	CT	Left lateralized extrusion of mineralized disk material at C3–C4, causing presumed compression of the left third spinal nerve root	C3–C4 2.1 mg/kg methylprednisolone 1 mg/kg bupivacaine	1.4 mg/kg tramadol q8 h 5.5 mg/kg gabapentin q12 h	Transient respiratory paralysis	Immediate resolution of pain and root-signature sings	2 weeks – pain free 4 years – pain free, medication free, no recurrence
10 years M Lhasa apso	1 week cervical pain, left forelimb root-signature signs	Meloxicam, diazepam	CT and MRI	Left lateralized extrusion of mineralized disk material at C3–C4 causing compression of the left third spinal nerve root	C3–C4 2.1 mg/kg methylprednisolone	3.2 mg/kg tramadol q8–12 h 6.4 mg/kg gabapentin q12 h	None	Immediate improvement	1 month – pain free 3 years – pain free, medication free, no recurrence
13 years SF mixed breed dog	2 weeks cervical pain, left forelimb root-signature signs	Methocarbamol, gabapentin, tramadol	MRI	Left lateralized extrusion of disk material at C5–C6 causing compression of the left fifth spinal nerve root	C5–C6 1 mg/kg methylprednisolone	5 mg/kg tramadol q12 h 10 mg/kg gabapentin q8–12 h 25 mg/kg methocarbamol q24 h	None	Marked improvement within 1 week	6 months – continued improvement, gabapentin PRN
11 years CM mixed breed dog	1 week cervical pain, tetraparesis, left forelimb root-signature signs	None	MRI	Left lateralized protrusion of disk annulus at C6–C7 causing compression of the left sixth spinal nerve root	C6–C7 1 mg/kg methylprednisolone	3–4.4 mg/kg tramadol q8 h 5.9–11.8 mg/kg gabapentin q12 h Prednisone 1.2 mg/kg q12 h	None	Improved pain control within 2 weeks	3 months – pain free, medications discontinued 1 year – pain free, medication free, no recurrence
6 years CM Dachshund	2 weeks cervical pain and left forelimb root-signature	Tramadol, methocarbamol, prednisone	MRI	Left lateralized extrusion of disk material at C6–C7 causing mild spinal cord compression and compression of the left sixth spinal nerve root	C6–C7 1 mg/kg methylprednisolone	2.5 mg/kg tramadol q8–12 h 10 mg/kg gabapentin q8–12 h 1 mg/kg prednisone q12 h	None	Mild improvement in hospital	3 weeks – moderate improvement 2–3 months – continued improvement and discontinuation of prednisone and tramadol; gabapentin PRN

**Figure 1:**
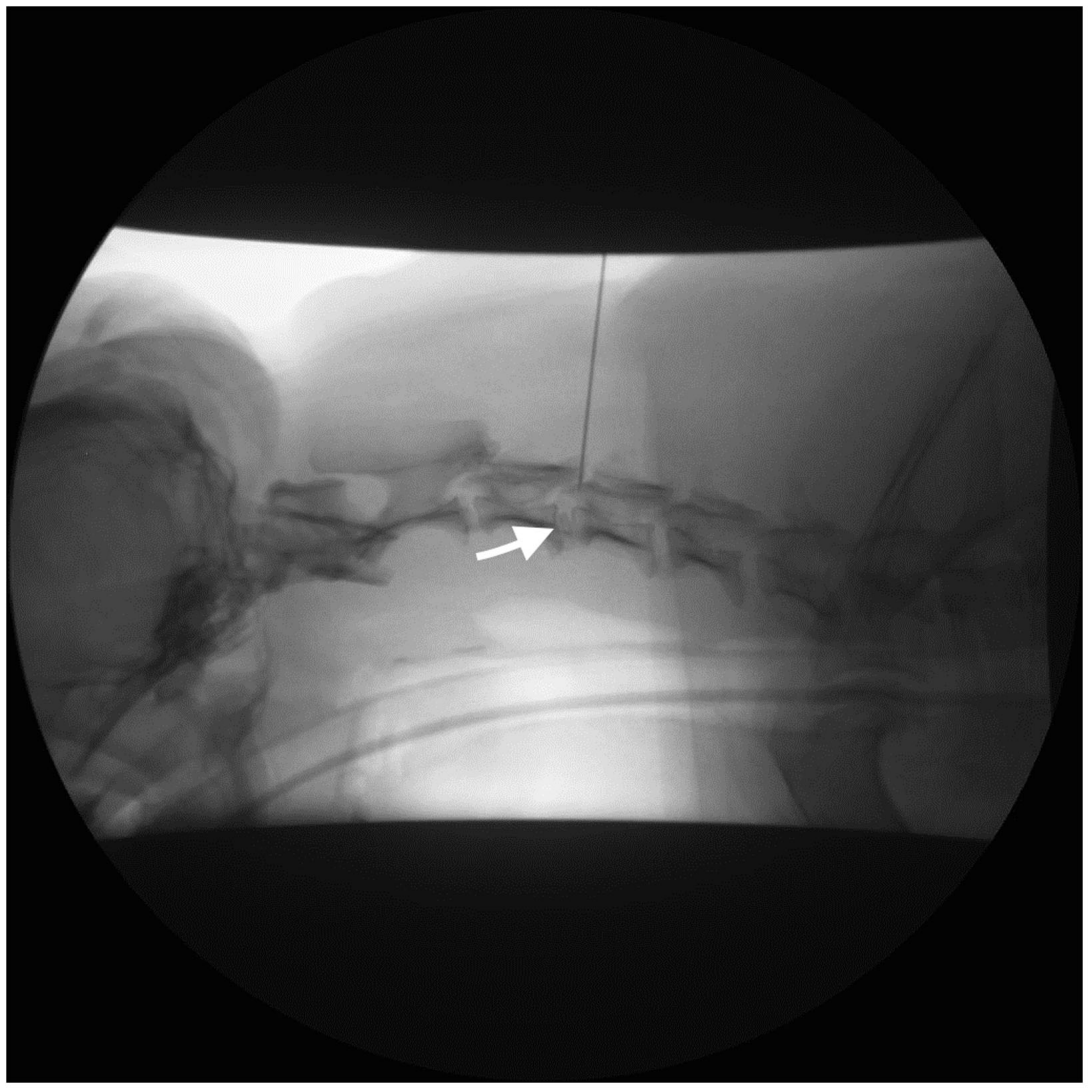
**Left lateral fluoroscopic projection of the cervical vertebral column (Case 1)**. A spinal needle is inserted dorsally at the level of the C3–C4 intervertebral foramen. Opacification of the C3-C4 intervertebral disk and foramen is also apparent (arrow).

For the perineural injection, each dog was positioned in lateral recumbency, affected side up, on the table of a digital fluoroscopy system (Shimadzu YSF-120 Digital R/F System, Shimadzu Medical Systems, Torrance, CA, USA). A 5 cm × 5cm area over the appropriate disk space on the lateral cervical spine was clipped and scrubbed. Fluoroscopy was used to guide needle placement (Figure 1). A 20 gage 1.5″ spinal needle was inserted from a dorsolateral direction until it contacted the articular process or lamina and then repositioned ventrally or caudally until superimposed with the target intervertebral foramen. Average procedural time was 25 min.

On admission to the VMCVM teaching hospital, clients provide written consent for teaching and investigational purposes via a standard treatment authorization form. All clients provided verbal consent for the treatment when therapeutic options were discussed. Each of these patients received follow-up phone calls as well and at the end of the phone conversation they were told that a case report was in the process and verbal consent was received.

### Case 1

A 10-year-old, neutered male Dachshund was presented for evaluation of severe neck pain that began 1 month earlier. Treatment prior to referral included deracoxib, tramadol, and cage rest. On presentation, physical and neurological examinations identified cervical pain and lameness of the left forelimb. Orthopedic examination was normal.

**Figure 2:**
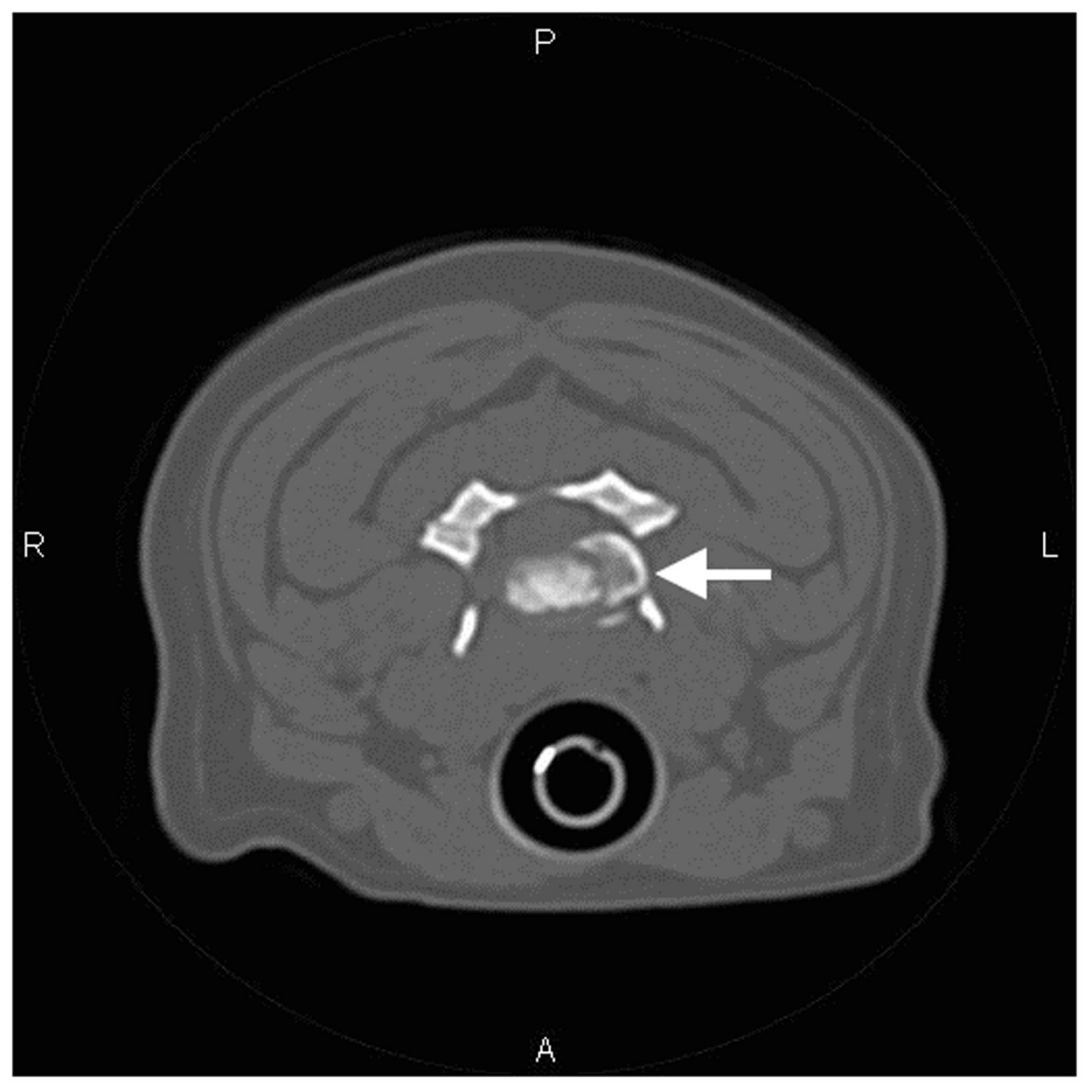
**Transverse CT image of the cervical spine at the level of C3–C4 (Case 1)**. Mineral-attenuating material is apparent in the left intervertebral foramen (arrow). There is also mineralization of the nucleus pulposus that remains *in situ*.

Computed tomography of the cervical spine was performed. At C3–C4, the intervertebral disk space was collapsed and a large amount of mineral-attenuating material was present in the left intervertebral foramen, displacing the perineural fat (Figure 2). Incidentally, *in situ* mineralization of the intervertebral disks was noted throughout the remainder of the cervical vertebral column.

The lateralized material at C3–C4 was thought to contribute to clinical signs due to compression of the left third spinal nerve, and its location suggested that it would not be accessible through a ventral slot approach. Interventional options included cervical hemilaminectomy or fluoroscopically guided perineural injection of local anesthetic (bupivacaine) and methylprednisolone. The client elected the latter.

Twenty milligrams of methylprednisolone (2.1 mg/kg) and 9.4 mg of bupivacaine (1 mg/kg) were injected into the left C3–C4 foramen. The patient was moved to the ICU after the procedure for recovery. Although the patient became increasingly alert, he initially failed to regain voluntary respiration. Mechanical ventilatory support was required for 30 min; later, the patient began breathing on his own, was weaned off the ventilator, and was extubated.

Immediate improvement in lameness was apparent while in the hospital. The patient was discharged with tramadol PO (1.4 mg/kg q8 h) and gabapentin PO (5.5 mg/kg q12 h) to be combined with cage rest and physical therapy. Fourteen days later at follow-up, the patient was doing well and was pain free. Tramadol was discontinued at that time, and the dose of gabapentin was reduced by half. By 3 months after surgery, all medications had been discontinued. Follow-up was obtained 4 years later when the patient was presented to the teaching hospital ophthalmology service for cataract evaluation. No recurrence of neck pain was reported by the client, and none was found on neurological examination.

### Case 2

A 10-year-old, intact male Lhasa apso was presented for evaluation of acute severe neck pain and left forelimb lameness that was unsuccessfully treated with medical management, including meloxicam and diazepam, for 7 days prior to presentation. On presentation, physical and neurological examinations identified left forelimb lameness, cervical pain, and mild lumbosacral pain. Orthopedic evaluation was normal.

Computed tomography of the cervical spine was performed, using the aforementioned protocol. The findings included intervertebral disk space narrowing at C3–C4 with an aggregation of mineral-attenuating material in the left intervertebral foramen and *in situ* mineralization of the intervertebral disk. Incidentally, there was also *in situ* mineralization of the intervertebral disk at C7–T1. Since no overt compressive spinal cord lesion was noted, cervical MR was performed to avoid administration of intrathecal contrast and provide a complete study. This revealed lateral disk extrusion into the left intervertebral foramen at C3–C4. The left third spinal nerve was not distinctly visualized in the foramen. There was also mild flattening of the ventral aspect of the cervical spinal cord at C2–C3 and C3–C4 secondary to hypertrophy of the dorsal longitudinal ligament and/or dorsal protrusion of the annulus fibrosis.

Treatment options were discussed and the client elected to have fluoroscopically guided perineural injection of 16 mg methylprednisolone (2.1 mg/kg) of the C3 left nerve root. Oral medications (gabapentin 6.4 mg/kg q12 h and tramadol 3.2 mg/kg q8–12 h) were continued.

Immediate improvement was apparent while in the hospital. The patient was discharged on the next day. The owner reported no pain at home following the procedure. All medications were discontinued 1 month after the procedure. Three years later, the patient had no recurrence of clinical signs reported by the owner via telephone follow-up.

### Case 3

A 13-year-old, spayed female mix breed dog was presented for evaluation of cervical and left forelimb pain beginning 2 weeks prior. She was receiving methocarbamol, gabapentin, and tramadol. On presentation, the patient was stiff in the forelimbs with lameness in the forelimb. Guarding was noted on cervical manipulation, and orthopedic evaluation was normal. Radiographs taken previously by the referring veterinarian revealed collapse of the intervertebral disk spaces at C4–C5 and C5–C6.

Magnetic resonance imaging showed a small amount of contrast enhancing material within the extradural space on the left ventral aspect of the vertebral canal at C5–C6. This material minimally displaced the left portion of the spinal cord dorsally and caused compression of the left fifth spinal nerve root within the C5–C6 intervertebral foramen. The intervertebral disk nuclei at C2–C3, C3–C4, C4–C5, C5–C6, and C6–C7 were hypointense on all sequences, consistent with *in situ* degeneration. Narrowing of the ventral subarachnoid space was present at C2–C3, C3–C4, C4–C5, and C6–C7, suggesting mild non-compressive intervertebral disk protrusions.

Treatment options were discussed and the owner elected to proceed with cervical perineural injection of the left C5 nerve root. Methylprednisolone (1 mg/kg) was injected into the perineural space. The patient was sent home on methocarbamol (25 mg/kg) q24 h, gabapentin (10 mg/kg) q8–12 h, and tramadol (5 mg/kg) q12 h as previously prescribed by the referring veterinarian for continued pain management.

Mild improvement in the patient’s clinical status was observed during hospitalization, most notable a decrease in cervical pain. The patient returned for follow-up 1 week later and the owner reported marked improvement in cervical and forelimb pain; findings were confirmed on physical exam. Six-month telephone follow-up revealed continued improvement in both cervical and forelimb pain. Gabapentin had been continued as needed for pain after prolonged activity, but all other medication had been discontinued.

### Case 4

An 11-year-old, neutered male mixed breed dog was presented for evaluation of an acute onset of severe neck pain and left forelimb lameness that worsened the week prior to presentation. He had received no treatment prior to presentation. Physical and neurological examinations identified mild tetraparesis and ataxia with left thoracic root-signature signs. Left forelimb lameness was noted with mild proprioceptive deficits in the left thoracic and pelvic limbs. The right forelimb was normal. Pain was apparent on cervical but not thoracolumbar manipulation. Orthopedic examination was normal. Findings were consistent with a lesion affecting the C6–T2 spinal nerves or spinal cord segments and T3–L3 spinal cord segments.

Magnetic resonance imaging of the cervical spine revealed that the nucleus of the intervertebral disk at C6–C7 was hypointense on all sequences, and there was mild broad-based dorsolateral protrusion of the C6–C7 intervertebral disk toward the left intervertebral foramen, compressing the left sixth nerve root. No spinal cord compression was noted.

Treatment options were discussed with the client. The client elected fluoroscopically guided perineural injection of methylprednisolone (1 mg/kg) into the area of the intervertebral foramen of C6–C7. Anesthetic recovery was uneventful.

Increased willingness to ambulate and improved voluntary range of motion of the head and neck were noted on exam the following morning; lameness was still present. The patient was subsequently discharged with gabapentin (5.9–11.8 mg/kg PO BID), prednisone on a tapering dose beginning at 1.2 mg/kg q12 h and tramadol (3–4.4 mg/kg PO q8 h). At the 2-week follow-up evaluation, the patient showed only mild pain during range of motion. Forelimb weakness and lameness were no longer evident. Prioprioception was improved in the left forelimb. T3–L3 signs were unchanged. Prednisone and tramadol were discontinued at this visit. Further telephone follow-up with the owner revealed that gabapentin was continued as needed for pain for 2–3 months then discontinued. The patient remained free from clinical signs of radicular pain but had succumbed to lymphoma 18 months later.

### Case 5

A 6-year-old, neutered male Dachshund was presented for severe cervical pain. Medical management, including tramadol, methocarbamol, and prednisone, was attempted for 2 weeks prior to presentation. Physical and neurological examinations identified lameness in the left forelimb and cervical pain. Mild postural reaction deficits and decreased spinal reflexes were noted in the left forelimb. Orthopedic examination was normal.

**Figure 3:**
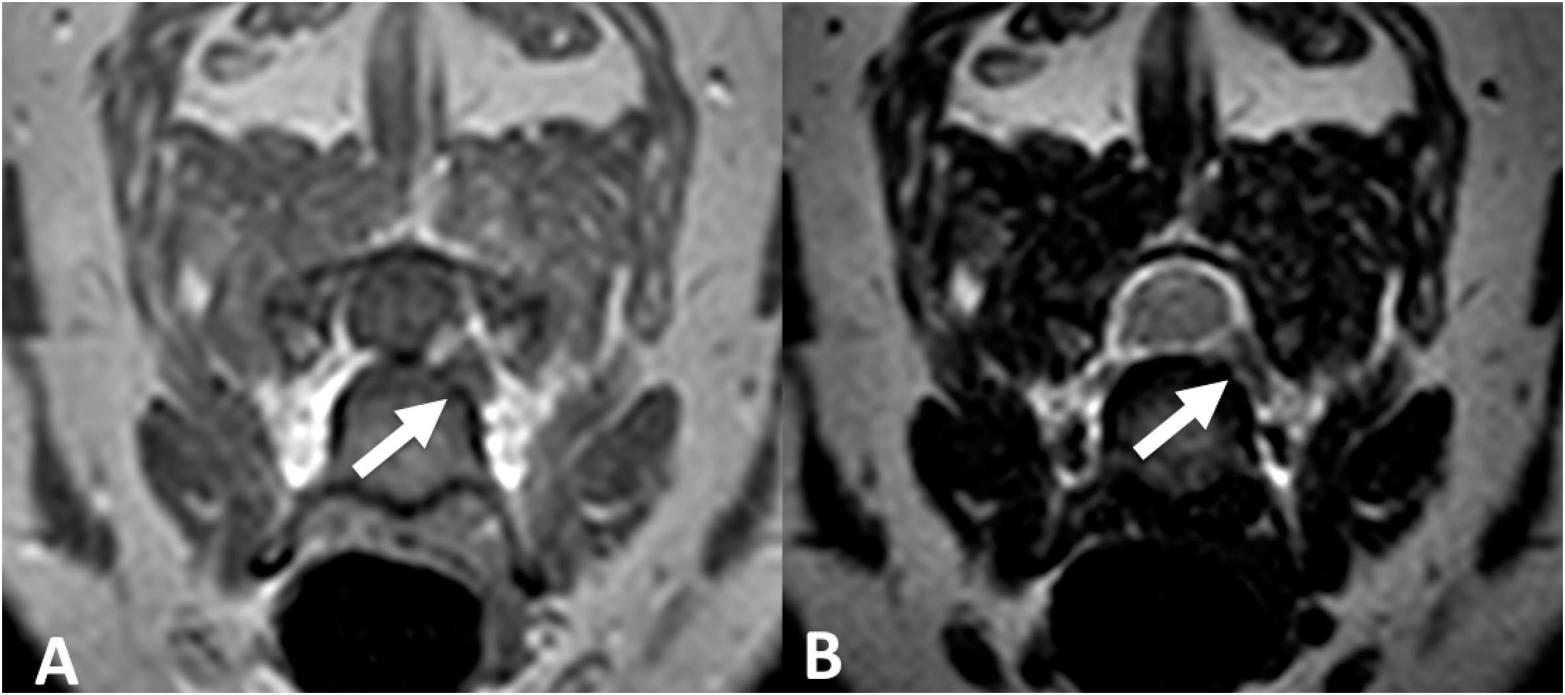
**Transverse T1-weighted (A) and T2-weighted (B) MR images of the cervical spine at the level of C6–C7 (Case 5)**. Hypointense extruded disk material is noted within the left intervertebral foramen on both sequences (arrows).

Magnetic resonance imaging of the cervical spine was performed. At C6–C7, there was an aggregation of material in the left ventral aspect of the vertebral canal and interverterbral foramen that was hypointense on T2W and STIR sequences with mild contrast enhancement on T1W sequence (Figure 3). This material resulted in mild left sixth spinal nerve root compression and minimal extradural spinal cord compression. Additionally, there was mild broad-based dorsal protrusion of the annulus fibrosis throughout the remainder of the cervical vertebral column, without spinal cord or nerve root compression.

Treatment options were discussed and the client elected fluoroscopically guided perineural injection of 5 mg methylprednisolone (1 mg/kg) at the C6–C7 foramen. Anesthetic recovery was uneventful.

Only mild improvement in pain was noted during hospitalization. The patient was discharged with gabapentin 50 mg (10 mg/kg) q8–12 h, tramadol 12.5 mg (2.5 mg/kg) q8–12 h, and prednisone 2.5 mg (1 mg/kg) q12 h the following day. The owner reported that over the next couple of months the patient continued to improve at home and both the tramadol and prednisone were discontinued by the owner and family veterinarian. Six months later, the clients were contacted by phone and reported that they administered gabapentin only as needed after intense exercise.

## Discussion

Cervical transforaminal epidural steroid injections are performed for treatment of radicular pain in people (11–14). Humans typically have multiple affected sites; it is advantageous to administer medications epidurally, rather than perineurally, allowing for more complete diffusion (11). This carries greater risk of neurological complications. Because of the focal nature of disease in cases identified here and the decreased risk of complications, perineural injections were considered. Because perineural injection does not alter the structural nature of the disease, rest for 4 weeks was also recommended. This was felt to be necessary to avoid acute recurrence associated with recrudescence of inflammation or herniation of additional disk material at the same site.

Four of five dogs had immediate improvement in clinical signs, the last case only showed mild improvement (Case 5). Three out of five dogs had long-term pain relief (18 months to 4 years) after perineural injection without continued administration of oral analgesics or recurrence of clinical signs. The two dogs that received on-going medical therapy (Cases 3 and 5) were administered gabapentin at the owner’s discretion.

One dog (Case 1) had significant complication of transient respiratory paralysis. Transient respiratory paralysis may have been caused by local anesthetic entering the epidural space and migrating cranially to result in depression of the respiratory centers at the craniocervical junction. Caudal migration of anesthetic and paralysis of the phrenic nerve could also explain this complication, although based on proximity this is considered less likely. Administration of a local anesthetic along with methylprednisolone was at the discretion of the clinician and based on results of Case 1 was not used in subsequent patients. Case 2 was affected at the same disk space (C3–C4), received steroid injection only, and experienced no complications. No other patient in this series experienced respiratory complications. Future studies are needed to evaluate the incidence of respiratory complications associated with injection technique, as well as to compare therapeutic benefit of steroid alone versus steroid in combination with local anesthetic.

In people, continued pain relief for 6–12 months following injection has been reported (12, 13). On-going relief of clinical signs may be attributable to the anti-inflammatory and anti-nociceptive properties of corticosteroids via decreased type C-fiber impulse transmission. C-fibers participate in chronic nerve pain, therefore blocking this transmission may result in prolonged relief of clinical signs (15). The anti-inflammatory properties of steroids may also contribute to membrane stabilization and reduction in nerve root swelling (16). Disk extrusions associated with Hansen type 1 degeneration in dogs are known to incite an inflammatory reaction that may play a role in the development of chemical meningitis or radiculitis (17). It is probable that steroids attenuate this reaction by inhibiting phospholipase A2 activity (18) and this may contribute to prolonged resolution of signs in some of our patients.

When single injections fail to produce sufficient long-term analgesia in people (<6–12 months), additional injections have been repeated with success (19, 20). Since no patient in this series re-presented for further evaluation and treatment, we cannot comment on use of repeat injections. However, failure to respond to perineural injection may be an indicator for surgical treatment.

Another theory explaining prolonged effects of epidural injection includes mechanical effects of the injection causing adhesion lysis, interruption of a reflex-sympathetic response, and membrane stabilization (21). Studies have also shown benefits of saline injection alone supporting the mechanical effects of the intervention and possible benefit from dilution of inflammatory mediators (22). None of our patients underwent repeat imaging or necropsy to assess mechanical effects or assess structural change. Lastly, inadvertent direct nerve injection could explain pain relief in some dogs; however, there was no sign of monoparesis or hyporeflexia supporting direct nerve injection.

No clients reported polyuria, polydipsia, alopecia, or weight gain to suggest significant systemic absorption of corticosteroid post-injection. However, the retrospective nature of this series precluded confirmation of suppression of the hypothyalamic–pituitary–adrenal (HPA) axis in all cases. Systemic absorption of epidural steroid remains a deciding factor when choosing patient suitable for perineural injection. An increase in blood pressure and blood glucose, as well as a decrease in cortisol and ACTH, has been demonstrated up to a week post-injection in people (23). Jacobs et al. (24) demonstrated marked suppression of plasma cortisol levels for 3 weeks in people after single lumbar extradural injection of methylprednisolone acetate (24). Similar absorption during perineural injection has not been extensively studied and further studies are needed to determine whether dogs intolerant of oral steroid medication or those on oral non-steroidal anti-inflammatory drugs may be better candidates for perineural administration of steroids.

Ultrasound has been used to identify vertebral anatomy with visualization of vascular tissue and individual nerve roots (25). Recent authors have described ultrasound-guided cervical intra-articular injections in canine cadavers, brachial plexus anesthesia in canine cadavers, and lumbar epidural in cadaver and live dogs (25–28). While ultrasound does not carry the risk of radiation exposure associated with fluoroscopy, it is more operator dependent and, at our institution, more expensive than fluoroscopy. Fluoroscopy was used to identify individual nerve roots for injection in the present study. CT offers the highest level of precision and accuracy but incurs a substantial cost compared to fluoroscopy and ultrasound (29). Pre-injection iodinated contrast could potentially improve technical precision in dogs with varied cervical morphology. However, in people, one prospective study found that the use of iodinated contrast did not affect the analgesic outcome (30). More rigorous assessment of risks of focal neuritis, seizures, and other neurological complications should be demonstrated prior to routine administration of contrast. Future studies are needed to compare the techniques in clinical patients.

## Concluding Remarks

Perineural steroid administration may provide effective analgesia in some cases of cervical foraminal intervertebral disk herniation. The procedure is inexpensive and requires short duration of anesthesia. Three out of five patients showed significant improvement immediately after perineural injection, while the remaining two required only intermittent dosing with gabapentin. One dog had complications. Prospective studies comparing administered medications, technique, and follow-up imaging are needed. Large prospective studies are needed to better assess complication rate. Patients with significant intervertebral disk material in the canal are unlikely to benefit from the procedure described and surgery should remain the primary consideration.

## Author Contributions

All authors made substantial contributions to the work and assisted in drafting and revising the submission. Each author has given final approval of the version to be published and is accountable for all aspects of the work.

## Conflict of Interest Statement

The authors declare that the research was conducted in the absence of any commercial or financial relationships that could be construed as a potential conflict of interest.
